# Temporal Structure and Complexity Affect Audio-Visual Correspondence Detection

**DOI:** 10.3389/fpsyg.2012.00619

**Published:** 2013-01-22

**Authors:** Rachel N. Denison, Jon Driver, Christian C. Ruff

**Affiliations:** ^1^UCL Institute of Cognitive Neuroscience, University College LondonLondon, UK; ^2^Helen Wills Neuroscience Institute, University of CaliforniaBerkeley, CA, USA; ^3^Wellcome Trust Centre for Neuroimaging, Institute of Neurology, University College LondonLondon, UK; ^4^Laboratory for Social and Neural Systems Research, Department of Economics, University of ZurichZurich, Switzerland

**Keywords:** multisensory, crossmodal, perceptual timing, perceptual correspondence, synchrony, information theory

## Abstract

Synchrony between events in different senses has long been considered the critical temporal cue for multisensory integration. Here, using rapid streams of auditory and visual events, we demonstrate how humans can use temporal structure (rather than mere temporal coincidence) to detect multisensory relatedness. We find psychophysically that participants can detect matching auditory and visual streams via shared temporal structure for crossmodal lags of up to 200 ms. Performance on this task reproduced features of past findings based on explicit timing judgments but did not show any special advantage for perfectly synchronous streams. Importantly, the complexity of temporal patterns influences sensitivity to correspondence. Stochastic, irregular streams – with richer temporal pattern information – led to higher audio-visual matching sensitivity than predictable, rhythmic streams. Our results reveal that temporal structure and its complexity are key determinants for human detection of audio-visual correspondence. The distinctive emphasis of our new paradigms on temporal patterning could be useful for studying special populations with suspected abnormalities in audio-visual temporal perception and multisensory integration.

## Introduction

When dealing with information from multiple senses, a key issue is to determine whether inputs in different senses are related or not, providing a multisensory version of the perceptual “correspondence problem” (Calvert et al., 2004; Spence and Driver, 2004; Shams and Beierholm, 2010). The role of *temporal* multisensory relations has often been studied for discrete pairs of events – one event in each of two modalities – via perception of simultaneity across modalities (Stone et al., 2001; Recanzone, 2003; Spence and Squire, 2003; Sugita and Suzuki, 2003; Fujisaki et al., 2004; Fujisaki and Nishida, 2005; Zampini et al., 2005; van Eijk et al., 2008) or via effects of stimulus (a)synchrony (Exner, 1875; Hirsh and Sherrick, 1961; Bertelson and Radeau, 1976; McGurk and MacDonald, 1976; Meredith et al., 1987; Munhall et al., 1996; McDonald et al., 2000; Shams et al., 2000; Watanabe and Shimojo, 2001). However, such approaches exclude the richness in temporal patterning that often exists for multisensory situations in real life (Arrighi et al., 2006). More complex natural stimuli – such as speech – apparently have longer audio-visual integration windows than simpler stimuli (Dixon and Spitz, 1980; Vatakis and Spence, 2006; Maier et al., 2011), providing an indirect hint that perception of audio-visual correspondence may be sensitive to the richness of temporal structure. But to our knowledge this has never been tested directly.

Previous studies have shown that for simple stimuli (such as visual flashes and auditory tones) arranged into extended streams, crossmodal integration is facilitated when the streams have matching as compared to unrelated event timing (Radeau and Bertelson, 1987). Particularly robust multisensory effects have been documented for streams that have irregular event timing; that is, a relatively complex temporal structure. Auditory and visual streams of this kind evoke larger BOLD responses in multisensory as well as primary sensory cortical areas when their temporal patterns coincide than when their patterns are unrelated (Noesselt et al., 2007). In addition, audio-visual integration of such streams can be shown to be statistically optimal when the streams have matching temporal patterns, but not when they have unrelated temporal patterns (Parise et al., 2012).

It is still unknown, however, what role temporal structure plays in these multisensory enhancements. In previous studies, auditory and visual streams with common temporal structure were synchronous or nearly synchronous. Therefore, it remains unclear whether common temporal structure alone can cue multisensory correspondence over a range of crossmodal lags. It is also unknown what aspects of temporal structure may be important for the perception of correspondence. We hypothesized, from a statistical perspective, that the complexity of shared temporal structure may enhance the perception of correspondence, because the more complex a temporal pattern, the less likely it should be to arise in separate modalities by chance.

Here we tested psychophysically whether humans can detect correspondence between rapid audio-visual event streams, based on their temporal *structure* alone, when importantly other temporal cues (such as synchrony) were kept constant across critical conditions. Similar methods have previously been used in a unimodal setting to provide evidence that common temporal structure leads to perceptual grouping of visual stimuli (Lee and Blake, 1999; Guttman et al., 2007). In addition, we systematically manipulated the temporal *complexity* of the audio-visual streams. We used information-theoretic measures to define and specifically manipulate different aspects of temporal complexity in our stimuli. This allowed us to characterize formal properties of complex temporal structure that may contribute to the perception of audio-visual correspondence. In the present set of experiments, we showed that sensitivity to multisensory correspondence can be enabled by shared temporal structure even across substantial lags and that temporal complexity increases correspondence sensitivity. Surprisingly, observers were sensitive to high-order aspects of temporal complexity – namely the predictability of inter-event interval sequences – over and above more basic forms of complexity based on stimulus occurrence or interval variability in the streams.

## Materials and Methods

### Participants

The 34 participants (aged 20–37 years) gave informed consent in accord with local ethical clearance. Two participants in Experiment 3 were excluded from analysis: one failed to comply with task instructions while the second reported significant perceptual fading of the visual stimuli during the experiment. Excluding the latter does not change the pattern of results. This left 32 participants (12 in Experiment 1, with 6 in each experimental group, 4 in Experiment 2, and 16 in Experiment 3). All were naïve to the purpose of the experiment (except for 1 of the 16 in Experiment 3, who was author R.N.D.; excluding her does not change the pattern observed). All reported normal or corrected visual acuity and normal hearing.

### Apparatus

In Experiments 1 and 2 (conducted at UCL), visual stimuli were displayed on a 19″ Mitsubishi Diamond Pro 920 CRT monitor with 1024 × 768 pixel resolution at a refresh rate of 60 Hz, viewed at a distance of 58 cm. Sounds were presented binaurally through Panasonic RP-HC 100 noise canceling headphones. In Experiment 3 (conducted at UC Berkeley), visual stimuli were displayed on a 19″ SONY GDM-500PS CRT monitor with 1024 × 768 pixel resolution at a refresh rate of 60 Hz, viewed at a distance of 64 cm. Sounds were presented binaurally through Sennheiser PX 200 headphones.

Presentation of all stimuli was controlled by custom-written software run in Matlab (Mathworks, USA), using the Cogent 2000 and Cogent Graphics toolboxes (University College London, UK) on a Dell Precision 650 computer in Experiments 1 and 2 and an HP Compaq dc7800 computer in Experiment 3. The computer monitor provided the only source of illumination in the testing room. Participants placed their chins in a rest during the experiment to constrain viewing distance.

### Stimuli

**Figure 1:**
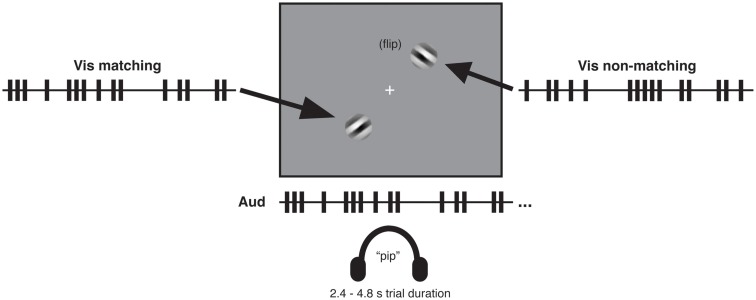
**Audio-visual matching task with temporally complex event streams**. Participants listened binaurally through headphones to one rapid stream of auditory tone pips (see illustrative train at center bottom) while viewing on a gray screen two streams of visual gratings in diagonally opposite quadrants. The gratings independently flipped between left- and right-tilted 45° orientations over the course of a trial. One of the two visual streams (the *matching* stream) corresponded to the auditory stream in its temporal pattern of orientation flips, which was either presented in synchrony with the auditory stream or delayed relative to that stream by specific temporal lags. The other visual stream (the *non-matching* stream) had a different temporal pattern of flips (see main text). After each trial (which lasted 2.4–4.8 s depending on the experiment and condition), participants reported which of the two visual streams had matched the auditory stream in temporal pattern. Example timelines (truncated here for brevity) of auditory and visual streams show auditory events (tone pips) and visual events (orientation flips) as trains of vertical bars. A key aspect of the procedure was that for all lagged conditions, the proportion of (coincidentally) synchronous audio-visual events was equated, see main text.

In all experiments, visual stimuli consisted of a centrally presented white fixation cross subtending 0.3° of visual angle and two square-wave grating patches presented in the lower left quadrant and upper right quadrant, respectively (see Figure 1). The grating patches were centered 3.8° of visual angle from the fixation cross. Each patch was created by applying a circular Gaussian mask to a 100% contrast black and white oriented square-wave grating with spatial frequency of 1 cycle per degree. The resulting patch subtended 3° of visual angle. At each screen position (i.e., lower left and upper right), two orthogonal patches oriented at 45 and −45° were presented in alternation, so as to create a dynamic visual stream within which a tilted grating flipped back and forth in orientation over time. All visual stimuli were presented on a medium-gray background. Auditory stimuli were sequences of 10 ms tone pips with a frequency of 2000 Hz and volume of 60 db.

### Procedure

#### Task

Participants listened to sequences of tone pips and simultaneously viewed two streams of visual events, in which oriented gratings flipped back and forth in orientation according to different temporal patterns in the lower left and upper right quadrants. Every trial started with presentation of a central fixation cross for 500 ms before the grating streams and auditory stream began.

On each trial, the temporal pattern of successive orientation changes for one of the grating streams matched the temporal pattern of the tone pips; this defined the *matching visual stream*. The other visual stream did not match the exact temporal pattern of the tone pips, thus providing the *non-matching visual stream*. The latter was equated to the matching visual stream for various aspects of its temporal statistics (see the Methods sections of the individual experiments below). The matching visual stream could either be presented in synchrony with the matching temporal pattern of the auditory stream (zero lag), or lagged relative to the auditory pattern by specific durations. Importantly, the matching and non-matching visual streams were generated according to the same stochastic point process, as explained below, and thus had comparable numbers of events that coincided with the tone pips for all non-zero lagged conditions (see also Lee and Blake, 1999; Guttman et al., 2007 for similar approaches in the visual domain). In a 2-alternative forced choice task, participants indicated via an unspeeded key press after the end of each trial which of the two visual streams had matched the auditory stream in temporal pattern.

Participants completed 40 trials per condition, as defined by lag (0, 1, 2, or 3 timebins); trial type (slower or faster presentation rate in Experiment 1; (pseudo)random or rhythmic in Experiments 2 and 3, see Methods sections below); and side of the screen on which the matching visual stream appeared (lower left or upper right). This resulted in a total of 640 trials. The experimental session was divided into 20 blocks, each containing two trials per condition in random order. Before the start of the experiment, participants also completed a practice block consisting of three trials per condition in random order. This practice block included feedback about accuracy of responses, but no feedback was given during experimental trials.

#### Generation of stimulus streams by a stochastic point process

**Figure 2:**
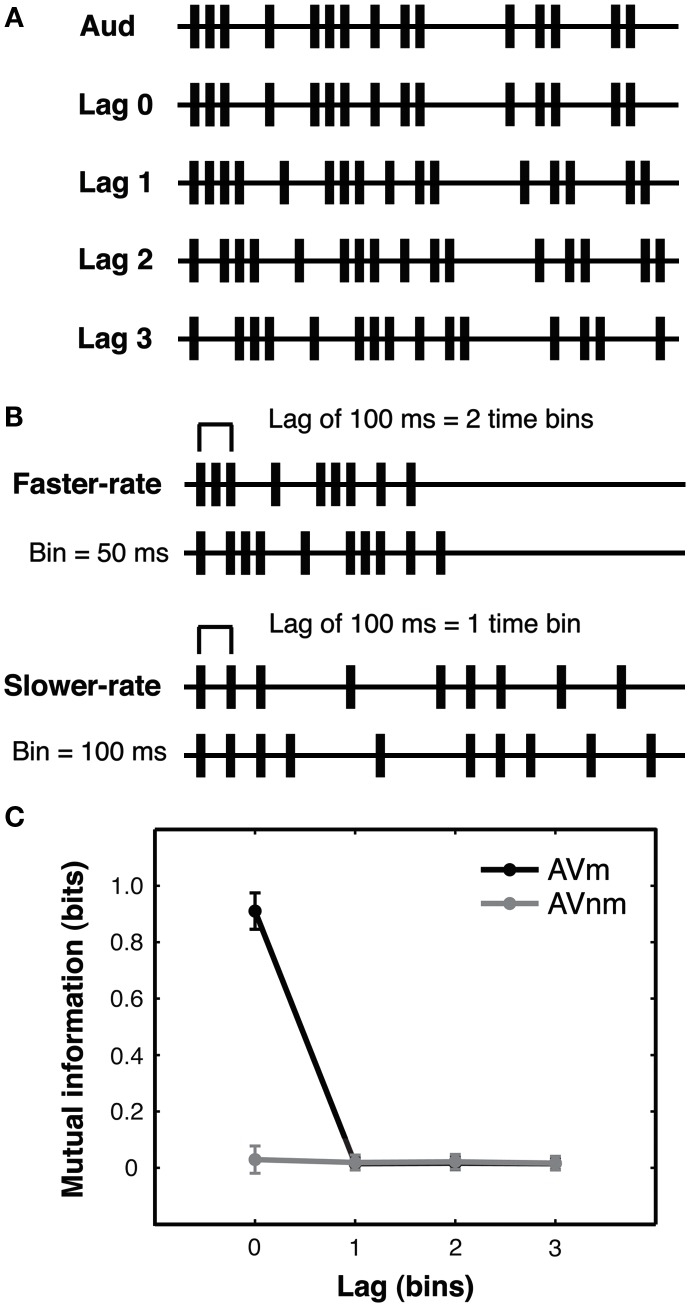
**Experiment 1: lagged stochastic event streams with varied presentation rate. (A)** Auditory streams (Aud) were generated using a stochastic point process with 1/3 probability that an auditory event would occur in each fixed-duration timebin. Matching visual streams (Lag 0–3) were identical to the auditory stream in temporal pattern and were either synchronous with the auditory stream (Lag 0) or could be delayed or advanced by 1–3 timebins (Lag 1–3, audition-leading-vision (AV) condition shown). Lagged events that exceeded the trial duration were looped back to the beginning of the stream to eliminate any gaps in the stimulus caused by lagging and to equate the number of events in the streams. Non-matching visual streams (not shown) were generated by shuffling the ISIs of matching streams (see text). All streams contained added initial and final events to equate stream onset and offset across streams. All lagged conditions were equated for the number of (coincidentally) synchronous events generated by the binned stochastic procedure, see main text. Example event streams are shown truncated here for brevity. **(B)** In the faster-rate condition, timebins were 50 ms and streams lasted 2.4 s, while in the slower-rate condition (presented at half that rate), timebins were 100 ms and streams lasted 4.8 s. Thus, a 100 ms time lag corresponded to a lag of two timebins in a faster-rate trial but only one timebin in a slower-rate trial. Preserving the number of possible events across rate conditions equated the information (entropy) carried by the two types of streams. Altogether, these stimuli allowed investigation of the effect of stream presentation rate for lags of identical duration and for streams with identical levels of information (see main text). Example auditory (upper stream) and matching visual (lower) stream timelines are illustrated here for each rate condition, truncated for brevity. **(C)** Mutual information between auditory and matching visual streams (AVm, black line) and between auditory and non-matching visual streams (AVnm, gray line) was calculated from first-order event entropy for a single event bin (see main text). Since this measure reflects simultaneous events, mutual information is present only for AVm streams at lag zero (i.e., perfectly synchronous streams). The plot shows means and standard deviations across trials, as calculated from the actual streams presented to the first subject of the AV experiment. Since faster-rate and slower-rate streams have the same number of bins, mutual information is the same for these stream types, and so we pool all streams for the analysis. This quantification of mutual information confirms that to achieve above-chance performance on lagged trials, observers must rely on different information than merely simultaneous events.

Irregular stimulus streams were randomly generated on each trial according to a stochastic point process (see Figure 2A and Guttman et al., 2007 for a related approach used there to create sets of visual sequences). Each trial period was divided into a series of equally spaced timebins. At the start of every timebin, an event in a given stream could occur with a probability of 1/3, resulting in sequences of events separated by variable inter-stimulus intervals (ISIs).

For every trial, the auditory stream was generated first and then appropriately shifted in time according to the lag condition to create the matching visual stream (Figure 2A). For example, at lag 2 in audition-leading-vision (AV) conditions, the matching visual stream would be delayed by 2 timebins with respect to the auditory stream. The non-matching visual stream for each trial was generated by shuffling the ISIs of the matching visual stream to create a new temporal pattern. Lagged matching visual events that extended beyond the fixed stream duration due to the lag were looped back to the corresponding positions in the beginning of the stream (thereby preventing any long “gaps” from emerging at the start of the trial). Moreover, the first and last timebins of each trial always contained an event in both modalities and at both visual locations for all conditions, thereby preventing participants from simply orienting to initial or terminal events.

The stochastic stream-generation procedure ensured that, for each experimental condition across all trials, stimulus statistics such as the mean and standard deviation of the ISIs were identical for all streams regardless of the lag condition. It also ensured that for all non-zero lag conditions, the proportion of synchronous audio-visual events were equated across such trials (see also Guttman et al., 2007).

#### Information-theoretic analysis of stimulus streams

As we were concerned here with the precise aspects of temporal information that cue temporal correspondence for human observers, we performed information-theoretic analyses of the stimulus streams to quantify the complexity (entropy) and relatedness (mutual information) of the streams.

##### Complexity

In Experiments 2 and 3, we manipulated specific types of temporal complexity while keeping others constant across conditions of comparison. Here we provide formal definitions of these types of complexity using information-theoretic measures. This further allowed us to calculate the entropy for our actual stimulus streams, confirming that these specific types of complexity were successfully manipulated or controlled in our experiments. The three types of complexity we considered were: (1) first-order event entropy (the presence or absence of an event at a given time point), (2) first-order ISI entropy (ISI variability), and (3) second-order ISI entropy (ISI sequence). To give a brief overview of our experimental manipulations of complexity: all conditions in Experiments 2 and 3 were matched on first-order event entropy; Experiment 2 manipulated first- and second-order ISI entropy; and Experiment 3 manipulated second-order while controlling first-order ISI entropy. The following describes each of these measures in detail.

The first type of complexity reflects the information contained in the presence or absence of a sensory event at a given time point. Each stream (which could be an auditory or a visual stream) is defined as a sequence of event bins in which either a visual or auditory change occurs (event = 1) or does not occur (event = 0). The entropy of such binary event streams thus represents the uncertainty about the occurrence of a given event within the stream, and we calculate the entropy of a bin *X* according to the following formula:

(1) $$H\left(X\right)=-\overset{n}{\underset{i=1}{\sum }}p\left(x_{i}\right)\log_{2}p\left(x_{i}\right),$$

where *x_i_* is the event state (0 or 1) possible for a bin, and *p*(*x_i_*) is the probability of event *x_i_* occurring, given the distribution of events in the stream. We call this *first-order event entropy*.

Since all streams (auditory, matching visual, and non-matching visual) in our experiments were stochastically generated with probability 1/3 of an event occurring in a given timebin, the first-order event entropy was equal for all streams in all experimental conditions, with a value of approximately 0.9 bits (for one bin), as confirmed by calculations using the actual streams presented to the first subject in each experiment.

The second type of complexity reflects temporal information arising from the temporal *intervals* present in a stream. Each stream, then, is a sequence of ISIs, which are simply the time differences between successive events in the stream. The entropy *H*(*X*) of an ISI *X* is calculated according to Equation 1, but now each *x_i_* is one of the unique ISI values in the stream. We call this *first-order ISI entropy*. A stream with more variable ISI duration, and thus more uncertainty about the duration of any given ISI, will tend to have higher first-order ISI entropy than a stream with lower ISI variability (in the extreme case, a perfectly periodic stream with only one repeated ISI). For our streams, first-order ISI entropy is identical across lag conditions (as confirmed by calculation) but can vary across experiments and is manipulated within subjects in Experiment 2, as described further in the Experiment 2 “Methods” section.

Finally, the third type of complexity reflects temporal information carried by the sequential structure of the stimulus streams. Observers sensitive to this type of complexity would be sensitive to *sequences* of ISIs and could exploit the information contained in such sequences. To take the simplest case, we consider each stream as composed of adjacent ISI pairs and calculate the *second-order entropy of the ISIs*. The entropy of an ISI pair *X* is *H*(*X*) (Eq. 1), where each *x_i_* is one of the unique ISI pairs present in the stream. Thus, a stream with only two unique ISIs but with those ISIs presented in random order would have higher second-order ISI entropy than a stream with two unique ISIs presented in repeated alternation. However, these streams would have equal first-order ISI entropy, since they both have two unique ISIs. In Experiment 3, we presented streams that were matched in first-order ISI entropy but differed in second-order ISI entropy, in order to assess whether such higher-order temporal structure information could influence judgments of multisensory relatedness in the matching task (see Experiment 3).

##### Relatedness

Within this information-theoretic framework, we could also calculate the mutual information between auditory and matching visual streams and between auditory and non-matching visual streams. We used this method to establish what pattern of performance we should expect from observers on the matching task if they only used information arising from simultaneous or near-simultaneous (within a bin duration) events in the auditory and visual streams. This would be the case, for example, if observers rely on synchronous auditory and visual events in order to judge audio-visual correspondence. It would also cover any case in which there is a predictable relationship between stream content at simultaneous time points in the two streams. Thus, just as for first-order event entropy, in order to calculate this *first-order mutual information of two event streams*, we take each stream to be a binary sequence with values of 1 in time bins where a sensory event occurred and values of 0 in time bins where no event occurred.

Mutual information *I* between simultaneous bins *X* and *Y* from two streams was calculated according to the formula:

(2) I(X;Y)=H(X)-H(X|Y),

where *H*(*X*) is the entropy of *X* (Eq. 1) and *H*(*X*|*Y*) is the conditional entropy of *X* given *Y*. Conditional entropy is given by the following equation:

(3) $$H\left(X|Y\right)=\overset{n}{\underset{i=1}{\sum }}\overset{m}{\underset{j=1}{\sum }}p\left(x_{i},y_{j}\right)\log_{2}\frac{p\left(y_{j}\right)}{p\left(x_{i},y_{j}\right)}.$$

Here, *X* and *Y* are simultaneous bins from two streams presented in the same trial; for example, *X* could be a bin in the auditory stream and *Y* could be a bin in the matching visual stream. Bin events *x* and *y* can each be either 0 or 1, so *n* = *m* = 2. The marginal probability that *y* takes on state *j* is given by *p*(*y_j_*) and the joint probability that *x* takes on state *i* at the same time as *y* takes on state *j* is given by *p*(*x_i_*, *y_j_*). Intuitively, mutual information quantifies the reduction in uncertainty about *X* when *Y* is known.

For each information-theoretic calculation, we used all of the actual streams presented to the first subject of each experiment (to ascertain that the relative values for the entropy and mutual information we had in mind when designing the point processes were actually expressed in the streams employed here). Mutual information for each stream pair (auditory/matching visual, auditory/non-matching visual) or entropy for each stream (auditory, matching visual, non-matching visual) were calculated for each trial, and means and standard deviations were taken across trials in each condition. Note that the *total* entropy/mutual information of the streams depends on both the information per stream unit (such as bin or ISI) and the number of units in the stream. Here we report information for a single stream unit to facilitate generalization of our calculations to other stream lengths, but note that in our experiments, stream duration (in such stream units) was always matched across conditions of comparison. We also only compare conditions with stream units of the same mean duration, so the quantities we report can also be converted to other temporal scales (e.g., bits/second) without loss of the relative levels of information across conditions.

## Experiment 1

We designed a situation where psychophysical sensitivity to audio-visual pattern correspondence could be assessed, despite constant levels of audio-visual synchrony across some of the conditions compared. Specifically, we used a single underlying stochastic point process (as in Guttman et al., 2007) to generate different event streams with rich temporal structure, by dividing time into “bins” of equal length and setting the probability of event occurrence (tone pips for auditory streams, grating orientation changes for visual) to a fixed value (1/3) for all timebins (see Figure 2A and Materials and Methods). On every trial in our 2AFC task, participants were presented with one such rapid stream of auditory tone pips and two streams of visual transients presented in opposite screen quadrants (Figure 1). One of the visual streams (the *matching* stream) perfectly corresponded to the auditory stream in its sequence of temporal intervals between successive events; but this stream could be presented with specific temporal lags relative to the sounds (see Materials and Methods). The other (*non-matching*) stream was a temporally shuffled version of this sequence, hence producing a different temporal pattern but with the same mean and standard deviations of inter-event intervals overall. On every trial, participants indicated by a button press whether the matching visual stream was in the left or right quadrant, providing a measure of sensitivity (*d*′) to temporal correspondence between auditory and visual streams. Importantly, the task of detecting temporal correspondences between auditory and visual streams was orthogonal to the temporal structure of the individual streams as well as their temporal proximity, allowing us to manipulate these factors and measure the resulting effects on sensitivity to temporal pattern correspondence.

This situation allowed us to test several issues, including: whether physical synchrony is essential for detection of audio-visual correspondences (lag 0 vs. all non-zero lags); the extent to which participants can detect correspondences at a range of temporal proximities (via the different lags); and how correspondence detection is affected by the rate of stimulus presentation (matched absolute time lags for streams of different rates). Crucially, our design allowed us to compare different non-zero lag conditions directly, because all such conditions had exactly the same proportion of synchronous events and comparable stream statistics (see Methods and Lee and Blake, 1999; Guttman et al., 2007).

### Methods

Streams were presented at two different rates in separate conditions, with all trial periods divided into a series of 48 equally spaced timebins. In the *slower-rate* condition, timebins were 100 ms in duration (4800 ms total stream duration), while in the *faster-rate* condition, timebins were 50 ms in duration (2400 ms total stream duration; Figure 2B). Thus the number of events (and hence total information in bits conveyed by each stream) was matched across the two presentation rates. For the faster-rate streams, ISI mean and standard deviation were 142 and 115 ms, respectively. For slower-rate streams, ISI mean and standard deviation were 287 and 232 ms, respectively. Note that these quantities are identical across the two rate conditions when ISI is measured in timebins. Both audition-leading-vision (AV) and vision-leading-audition (VA) conditions were tested, in separate groups of participants (*N* = 6 for each group), in order to examine possible modality order asymmetries.

Calculations of the first-order event mutual information (see Materials and Methods) confirmed that mutual information was present between the auditory and matching visual streams only at lag 0, when an event in the auditory stream perfectly predicted the (synchronous) event in the visual stream. At all non-zero lags, mutual information between auditory and visual streams was zero for both matching visual and non-matching visual streams (Figure 2C). For all non-zero lags, an observer sensitive only to event coincidence would thus have no information available that could be used to match the temporal streams. Note that coincident events were defined as events that appeared within the same short time window of a timebin (see Materials and Methods), so this analysis also rules out matching based on auditory and visual events occurring close together in time but not necessarily in perfect synchrony (as commonly occurs in the real world).

### Results

#### Shared temporal structure is a reliable cue for audio-visual correspondence, with no special role for physical synchrony

**Figure 3:**
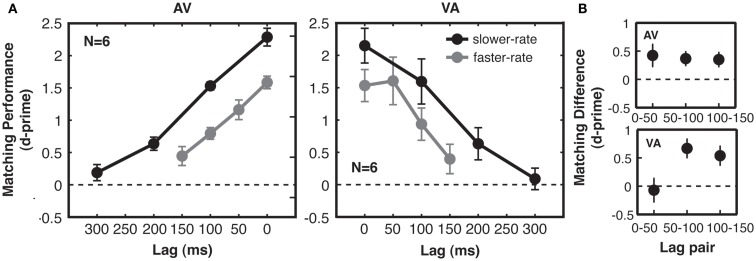
**Experiment 1: correspondence sensitivity based on shared temporal structure. (A)** Matching performance (cross-modal correspondence sensitivity, indexed by 2AFC *d*-prime) was above chance for lags of up to 200 ms. Performance decreased linearly with cross-modal lag, with no special (i.e., no supra-linear) benefit for synchronous streams at lag 0, as compared with the other lags. Performance was higher for slower-rate (black) trials than for faster-rate (gray) trials, but the effect of time lag on performance for the two types of trials was similar. For slower-rate trials, performance was similar when auditory streams preceded matching visual streams (AV subjects, left) and when auditory streams followed matching visual streams (VA subjects, right). **(B)** Quantification of modality order effect for faster-rate trials. Differences in matching performance between adjacent lag pairs were taken for each subject. All error bars give standard error of the mean between subjects.

In a three-way ANOVA including all *d*′ data from Experiment 1, we found no significant interactions between the within-subjects factors (presentation rate and lag) and the between-subjects modality order factor (AV vs. VA), so we turn to the effects of the within-subjects manipulations on *d*′. We found, first, that correspondence sensitivity decreased with increasing lag between matching auditory and visual streams [*F*(3, 30) = 86.3, *p* < 0.001, main effect of lag in the three-way ANOVA; see Figure 3A]. This was true for both the faster-rate [*F*(3, 30) = 33.4, *p* < 0.001] and slower-rate [*F*(3, 30) = 108.8, *p* < 0.001] conditions when considered individually. However, it was clearly not the case that participants could only perform the matching task when the related auditory and visual streams were perfectly synchronous. Rather, participants detected correspondence above chance for lags of up to 200 ms [pooling across AV and VA groups, *t*-test of slower-rate, lag 2 (200 ms), compared to zero, *t*(11) = 5.0, *p* < 0.01 Bonferroni-adjusted for multiple comparisons against zero; Figure 3A]. Note that for non-zero lag conditions, audio-visual matching was not determined by synchronous events alone: performance improved as the temporal proximity between matching stream patterns increased, even though these conditions had identical (chance) levels of audio-visual synchrony and identical levels of mutual information when considering event coincidence as the relevant temporal cue used to make the judgments (see Materials and Methods, Figure 2C, and also Lee and Blake, 1999; Guttman et al., 2007).

Moreover, physical synchrony (zero lag) for all matching events did not enhance correspondence sensitivity over and above that predicted by performance at the various non-zero lags. A special enhancement for synchronous compared to lagged streams would produce a deviation from linearity over the lags tested in the form of an extra performance benefit at lag 0. To statistically test for the presence of such an improvement, we compared quadratic and linear model fits to the data and found a no-greater-than-linear relationship between sensitivity and lag [*F*(1, 10) = 153.5, *p* < 0.001; see Figure 3A], with no significant explanatory power gained by adding a quadratic term [*F*(1, 5) = 0.14, *p* = 0.71, n.s.]. This confirms that the zero lag condition did not deviate from the level of performance that would be predicted based on the pattern for the three non-zero lags. Moreover, the increase in *d*′ between lag 1 and lag 0 (reflecting any behavioral benefit due to perfect synchrony) was no greater than the corresponding increase from lag 2 to 1 (in fact, any trend was the opposite way; see Figure 3). These results indicate that streams with perfect synchrony do not behave qualitatively differently from lagged streams, once temporal proximity is taken into account as a linear variable. Over the range of lags tested here, performance was well-described by a linear function of lag (mean norm of residuals to linear fits across the four conditions = 0.2 *d*′ units). However, performance for longer lags than tested here would asymptote to a *d*′ of zero (chance performance), so a bell-shaped function is likely a better characterization of performance in this task across all possible lags.

#### Sensitivity of matching performance to stream rate and modality order

If detection of related structure in auditory and visual streams relies on multisensory integration mechanisms, then matching performance should reproduce findings from synchrony and temporal order judgment tasks. We assessed effects of stream rate and modality order, which have been studied using these tasks. From the stream rate manipulation, we observed higher correspondence sensitivity for the slower-rate streams than for the faster-rate streams (Figure 3A). At the two lag points for which absolute crossmodal delays were matched in the two rate conditions (0 and 100 ms), correspondence sensitivity for slower-rate streams compared to faster-rate streams was greater by 0.67 in units of *d*′. This main effect of rate was significant in a planned three-way ANOVA with the same factors of rate, lag, and modality order, but now including only the 0 and 100 ms levels of lag [*F*(1, 10) = 50.2, *p* < 0.001]. There was no interaction between lag and rate [*F*(1, 10) = 0.2, *p* = 0.70, n.s.]. Indeed, the two rate conditions were well-matched quantitatively in the effect of absolute time lag on performance (linear fit to group data, mean across AV/VA groups: faster-rate slope = −0.0079 *d*′ units/ms of lag; slower-rate slope = −0.0072 *d*′ units/ms of lag), showing that the dominant effect of the rate manipulation was an overall change in correspondence sensitivity.

Second, while performance in the AV and VA groups was similar overall, our data suggested a modality order difference at lags of 50 ms. Matching performance on AV faster-rate trials showed a linear fall-off with increasing lag, with equal declines between all adjacent lag pairs (Figure 3B). However, VA matching performance showed a different pattern, with equivalent performance for 0 and 50 ms, followed by a decline between subsequent lag pairs (Figure 3B). We tested these effects for fast-rate streams using a two-way ANOVA of *d*′ differences between adjacent lags, with factors of modality order and lag pair. The interaction between modality order and lag pair was short of statistical significance [*F*(2, 20) = 2.5, *p* = 0.10]. However, enhanced correspondence sensitivity when vision leads audition by 50 ms is consistent with typically reported points of subjective simultaneity (see Discussion). We did not observe a V-first advantage for lags of 100 ms (Figure 3A).

## Experiment 2

Our results indicate that perception of audio-visual correspondence for rapid streams can exploit relationships between temporal patterns in the two modalities. This suggests that the quality of temporal information contained within such patterns should influence correspondence sensitivity. We next tested this directly by manipulating the temporal *complexity* of event patterns in the stimulus streams.

### Methods

We varied stream complexity according to two possible types of temporal information to which observers may be sensitive, quantified by the first-order and second-order entropies of the ISI distributions (see Materials and Methods). First-order ISI entropy reflects the entropy of the distribution of ISI durations in each stream. Second-order ISI entropy reflects the entropy in the distribution of ISI transition probabilities – that is to say, the variability in transitions between ISIs of specific durations and thus the predictability of the *sequence* of ISIs. In Experiment 2, we designed streams that differed in both their first-order and second-order ISI entropies.

**Figure 4:**
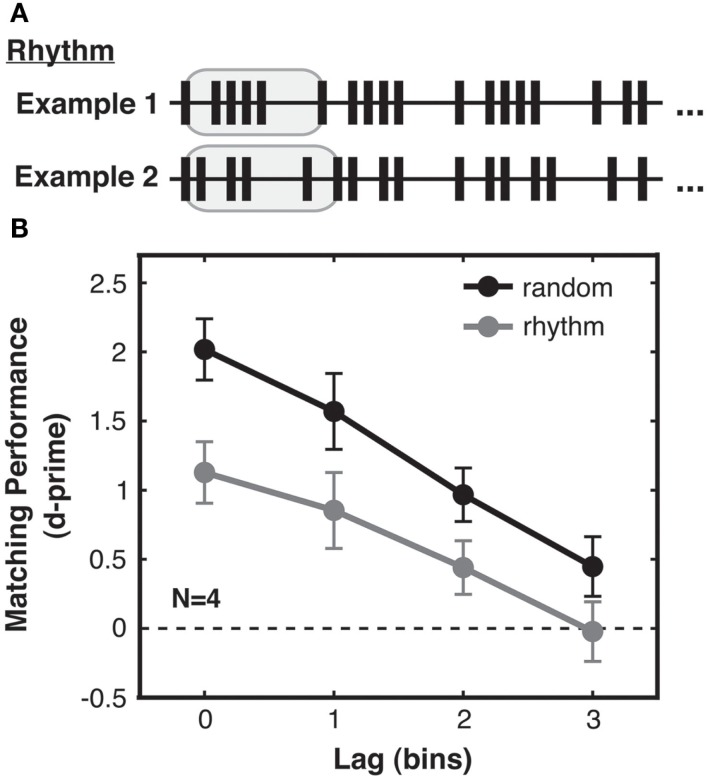
**Experiment 2: pattern complexity increases sensitivity to audio-visual correspondence. (A)** Rhythmic streams were created by first generating (as in Experiment 1) a random stream “bar” lasting five ISIs (highlighted in gray), and then repeating that bar to fill the trial duration. Rhythmic streams consequently had lower temporal pattern complexity than random streams, due to the redundancy arising from bar repetition. This is quantified by differences in both first-order and second-order ISI entropy in the streams resulting from the ISI distributions of random and rhythmic streams (see main text). Examples of two rhythmic streams generated on different trials are shown (truncated for brevity). **(B)** Matching performance was better for random streams (black) with higher pattern complexity, compared to performance for rhythmic streams (gray). Performance for both stream types decreased in a similarly linear fashion with lag. Standard error of the difference between random and rhythmic conditions is shown for each lag.

Under this framework, we generated two types of streams, “random” and “rhythmic,” which differed in the complexity of their temporal structure. In the random condition, the streams were generated stochastically as in Experiment 1. These streams were highly irregular and unpredictable, and thus had high pattern complexity [mean first-order ISI entropy = 2.9 bits (SD across trials = 0.2), mean second-order ISI entropy = 3.9 bits (SD 0.2)]. In contrast, rhythmic streams (Figure 4A) contained several repetitions of a five-ISI sequence, which we will call a “bar” in analogy to a bar of music. This bar was generated according to the same stochastic point process as used for random streams, but was then cycled repeatedly to fill the trial duration (different bars and thus different ISIs were generated for each trial; see Figure 4A). The matching visual stream was generated as in Experiment 1, whereas the non-matching visual stream consisted of a new rhythm based on a shuffled version of the bar used to create the auditory stream. The non-matching bar comprised the same five ISIs as for the original bar, but constrained to be in a different order within that bar, and was then cycled repeatedly to fill the trial duration. Since in all other respects the same stochastic process was used to generate random streams and rhythmic bars, random and rhythmic streams had similar mean and standard deviation of ISIs across trials (over all trials: ISI mean = 186 ms, ISI SD = 147 ms). However, rhythmic streams had lower pattern complexity, as they were more regular [mean first-order ISI entropy = 2.3 bits (SD 0.4)] and repetitive [mean second-order ISI entropy = 3.0 bits (SD 0.5)] than random streams.

The rate of the streams was fixed with 66.67 ms timebins and the total duration for each stream set to 4000 ms. Possible lags were again 0, 1, 2, or 3 timebins. In lagged trials, auditory events always preceded their corresponding visual events.

### Results

#### Pattern complexity increases sensitivity to audio-visual correspondence

We found that the type of temporal structure present in the stimulus streams significantly influenced sensitivity to audio-visual correspondence. Specifically, participants were better at detecting correspondences for random than for rhythmic streams [2-way ANOVA with factors complexity and lag, main effect of complexity: *F*(1, 3) = 11.84, *p* < 0.05; Figure 4B], demonstrating that higher pattern complexity led to improved performance. We also replicated the findings from Experiment 1 that correspondence sensitivity decreased with lag [main effect of lag: *F*(1, 3) = 15.06, *p* = 0.001], and that this relationship was again linear [*F*(1, 3) = 23.35, *p* < 0.05] with no significant contribution from a quadratic term [*F*(1, 3) = 0.17, *p* = 0.78, n.s.]. No interaction was found between pattern complexity and lag [*F*(1, 3) = 1.67, *p* = 0.24, n.s.]. Pattern complexity thus increased correspondence sensitivity without leading to fundamentally different processing strategies.

## Experiment 3

Experiment 2 showed that audio-visual matching performance is enhanced for random vs. rhythmic rapid streams, suggesting that complexity of temporal structure aids detection of audio-visual correspondence. However, the random and the rhythmic streams in Experiment 2 differed in both interval variability (first-order ISI entropy) and in temporal predictability of ISI durations (second-order ISI entropy), leaving it unclear which of these two aspects of complexity impacted performance. In Experiment 3, we tested whether increased second-order ISI entropy alone is sufficient to enhance correspondence detection by comparing audio-visual matching performance for random and rhythmic streams that now differed only in temporal predictability for the sequence of intervals, not in terms of interval variability on any single trial.

### Methods

**Figure 5:**
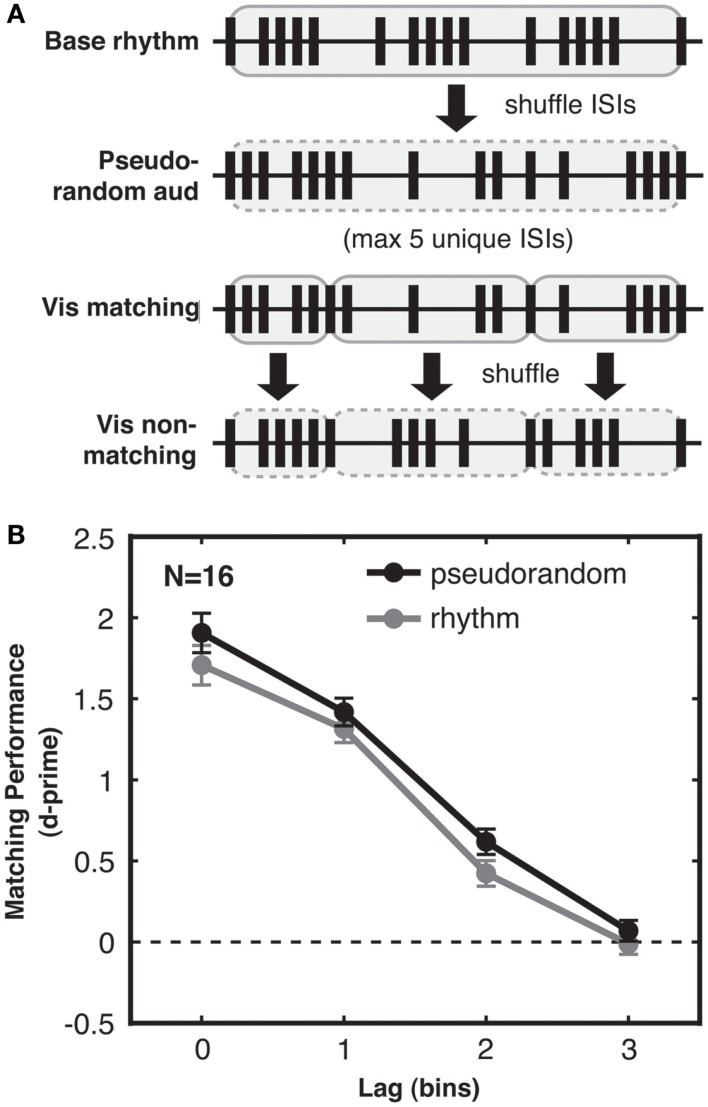
**Experiment 3: audio-visual correspondence sensitivity depends on sequence complexity, not merely ISI variability. (A)** Generation of pseudorandom streams. From top to bottom: first, a base stream was generated as for rhythmic trials. All ISIs in this stream (highlighted in gray with solid outline) were then shuffled to create the pseudorandom auditory stream (shuffled ISIs highlighted in gray with dashed outline). Accordingly the distribution of ISIs was identical for pseudorandom and rhythmic streams. The matching visual stream (shown at lag 0 for simplicity here) was then divided into five-ISI bars (each highlighted in gray with solid outline). Each of these bars was then internally shuffled to generate the non-matching visual stream (shuffled bars highlighted in gray with dashed outline). Hence the relationship between the ISIs of the non-matching stream and the ISIs of the accompanying auditory and matching streams were the same for pseudorandom and rhythmic streams even at the bar-length timescale. In an information-theoretic analysis, this stream generation procedure resulted in pseudorandom and rhythmic streams with ISI distributions that had identical first-order entropies but still differed in second-order entropy, with more information in pseudorandom streams due to their more variable ISI transitional probabilities (see main text). **(B)** Matching performance was enhanced for pseudorandom streams (black) compared to rhythmic streams (gray), with only sequence complexity differing between streams in the two conditions, and higher complexity leading to better performance. Standard error of the difference between pseudorandom and rhythmic conditions is shown for each lag.

We generated “pseudorandom” rather than entirely random streams so as to match them more closely to rhythmic streams in specific ways. First, pseudorandom streams in Experiment 3 were designed to match rhythmic streams even in terms of ISI variability *within* each trial, and not only across trials. To achieve this, pseudorandom streams were created by first generating rhythmic bar-repeat streams (as in Experiment 2), then randomizing the order of their ISIs over the full trial duration (Figure 5A). Like the rhythmic streams, the resulting pseudorandom streams had at most five unique ISIs repeated an equivalent number of times, but now rearranged to form a less predictable pattern than that of rhythmic streams. Thus, on any given trial in Experiment 3, only the random temporal *order* of the ISIs, not the diversity of ISI durations, made random streams more complex than rhythmic streams. Second, the pseudorandom non-matching visual streams were created by local shuffling of the ISIs within each of the five-ISI bars. Thus, for both the rhythmic and pseudorandom conditions, a bar of five ISIs in the matching visual stream was always comparable to a shuffled bar of the same five ISIs in the non-matching visual stream. Finally, a random lag (from 0 to 3 bins) was introduced to the non-matching visual stream in both rhythmic and pseudorandom trials. This prevented above-chance synchronicity between rhythmic auditory and non-matching visual streams, which might otherwise have arisen if the use of the same five ISIs in distinct rhythmic patterns led to systematic relations between events in the two streams. Taking all of these issues into account, the highly controlled stimuli of Experiment 3 were designed to match pseudorandom and rhythmic streams in every respect except for complexity of temporal structure, as quantified using the distribution of ISI transitions. Specifically, mean first-order ISI entropy for pseudorandom and rhythmic streams was matched at 2.3 bits (SD 0.3 for pseudorandom, 0.4 for rhythmic), while mean second-order ISI entropy was greater for pseudorandom streams (3.6 bits, SD 0.3) than for rhythmic streams (3.0 bits, SD 0.5). ISI mean and standard deviation were matched for pseudorandom and rhythmic streams at 181 and 140 ms, respectively. As in Experiment 2, all streams were 4000 ms in duration with 66.67 ms timebins, and possible lags were 0, 1, 2, or 3 timebins.

### Results

#### Enhancement of audio-visual correspondence detection due to pattern complexity reflects sequence information and not only ISI variability

With these strongly controlled stimuli, we still found a highly reliable (albeit numerically smaller) effect of temporal pattern complexity on audio-visual matching performance, with higher correspondence sensitivity for pseudorandom compared to rhythmic streams [2-way ANOVA with factors complexity and lag, main effect of complexity: *F*(1, 15) = 9.038, *p* < 0.01; see Figure 5B]. Again, we saw a strong main effect of lag [*F*(3, 45) = 187.906, *p* < 0.001] and no interaction between lag and complexity [*F*(3, 45) = 0.483, n.s.]. This replicates Experiment 2 and further demonstrates that the benefit in matching performance for complex streams is not solely due to higher variability in ISIs within trials (Experiment 2 had already ruled this out between trials) nor any other statistical difference in the stimuli. Hence a benefit in crossmodal matching performance genuinely results from the higher complexity of the temporal *sequence* within (pseudo)random streams as compared to rhythmic streams.

## Discussion

Using tight statistical control of complex audio-visual streams, our experiments revealed that detection of audio-visual correspondence is possible across substantial lags, depends on temporal complexity (in an information-theoretic sense), and therefore is critically tied to the temporal structure rather than merely the temporal coincidence in extended streams. Our findings and their implications take us beyond traditional perspectives on the contribution of timing to detection of audio-visual relations. Previous studies using simple auditory and visual stimuli have often focused on temporal relations between discrete pairs of events (e.g., one in each modality Shams et al., 2000; Stone et al., 2001; Watanabe and Shimojo, 2001; van Eijk et al., 2008) rather than rapid extended streams as here, and had suggested possible “time windows” within which auditory and visual events might be treated as related. Such suggested time windows typically varied substantially between studies – in particular, suggested multisensory time windows appeared to be longer for complex ecological stimuli (such as audio-visual speech) than for simpler auditory-visual pairings such as flashes and beeps (Dixon and Spitz, 1980; Vatakis and Spence, 2006; Maier et al., 2011). These findings have been taken to indicate variability in the time windows for multisensory integration (Spence and Squire, 2003); however, the underlying temporal mechanisms supporting longer integration windows for some stimuli than for others remain poorly understood. Here we found that people can detect audio-visual correspondence even for lags of 200 ms (Experiment 1) when using simple flashes and beeps, provided these appeared in extended, temporally structured streams. As a reference, the window for perception of audio-visual simultaneity with single discrete pairs of auditory and visual events (rather than matching of patterned streams as here) is less than 100 ms, and usually within 50 ms (Hirsh and Sherrick, 1961; Kristofferson, 1967; Zampini et al., 2005; Vroomen and Keetels, 2010). This indicates that even for simple, non-semantic stimuli, detection of audio-visual correspondence across substantial lags can be enabled by rich temporal structure.

Our results also indicate that exact temporal coincidence (0 ms lag) between visual and auditory events may not play a special role in facilitating correspondence detection between auditory and visual streams. Performance at zero lag in Experiment 1 was (linearly) predictable from performance at non-zero lags, indicating no unique benefit for zero lag, but rather simply a general (no-greater-than-linear) improvement at reduced lags. Moreover, detection of audio-visual correspondence differed systematically between the various non-zero lags, even though those all shared exactly the same proportion of crossmodal temporal coincidences (see Materials and Methods and Lee and Blake, 1999; Guttman et al., 2007).

Because our stream-matching task did not require judgments about the relative timing of the auditory and visual stimuli, our results reflect detection of multisensory relatedness as opposed to determinations of precise timing relationships between events in the two modalities. Assessing whether or not multisensory stimuli are related is arguably closer to the ecological demands of solving the multisensory correspondence problem than detecting small differences in the onset times of auditory and visual stimuli, as required in synchrony and temporal order judgment tasks. Still, our results agree well with previous findings from the timing judgment literature. For example, increasing the presentation rate of the audio-visual streams led to decreased correspondence sensitivity. This is consistent with previous experiments using periodic audio-visual event streams, in which synchrony judgments were more difficult for higher frequency streams (Fujisaki and Nishida, 2005, 2007). One limitation of these previous experiments was that crossmodal lag co-varied with event frequency, so it was not clear which of these factors was driving the changes in performance. Our study matched crossmodal lag at critical time points in the faster-rate and slower-rate conditions and still observed reduced correspondence sensitivity for faster-rate streams. We controlled for the number of stream events in the two rate conditions, such that the number of possible audio-visual comparisons (and thus the information contained in the two streams) was identical across conditions.

While the general effect of increasing presentation rate is similar in our study and previous studies, the specific stimulus rates supporting successful task performance differ across studies. Fujisaki and Nishida (2005, 2007) concluded that the threshold for synchrony detection was around 4 Hz. Our results showed correspondence detection well above chance for the faster-rate streams, even when lagged, although these streams had an average presentation rate of 7 Hz. This difference between studies could arise from differences in task (synchrony detection vs. correspondence detection), differences in stimulus temporal structure (periodic vs. stochastic event streams), or a combination of these factors. Evidence that temporal structure is an important factor comes from a previous finding (Fujisaki and Nishida, 2007) that subjects can easily detect asynchronies of 250 ms (compared to 0 ms) in stochastic audio-visual event streams for average event rates of 13 Hz. Although differences in methodology between synchrony judgment experiments that used periodic vs. stochastic event streams prevent their results from being compared directly, these studies and ours establish constraints on the mechanisms of temporal perception of multisensory stimuli that can guide future investigations.

We also observed a trend for a modality order effect, in which correspondence sensitivity was reduced for AV lags of 50 ms compared to 0 ms but was preserved for VA lags of 50 ms. Because this effect did not reach significance, our interpretation must be speculative. However, this relative performance enhancement when vision precedes audition matches findings from many previous studies in which single visual stimuli are perceived to be simultaneous with auditory stimuli occurring 20–60 ms later (see van Eijk et al., 2008 for a review). Our study raises the possibility, which could be targeted in future experiments, that vision preceding audition on this timescale also leads to a behavioral advantage for detecting audio-visual relatedness in addition to affecting judgments of precise temporal coincidence or order. Such a behavioral advantage may be related to electrophysiological findings that audio-visual interactions in auditory cortex are strongest when a visual stimulus precedes its corresponding auditory stimulus by 20–80 ms (Kayser et al., 2008; Thorne et al., 2011).

Even though findings from our correspondence detection task share notable similarities with those from previous temporal order and synchrony judgment tasks, comparison of results across tasks is also limited by task differences. The correspondence detection task measures perception of temporal structure relationships across modalities, while the other tasks measure perception of precise timing relationships. In addition, the correspondence detection task is a 2-alternative forced choice task, in which one of two visual streams is related to a single auditory stream. Synchrony and temporal order judgment tasks typically present a single visual and auditory stream. The presence of competing visual streams likely increases the difficulty of our task, as would be expected from findings that distracting visual information impairs audio-visual synchrony detection (Fujisaki and Nishida, 2007). In future work, it will be informative to elicit synchrony and temporal order judgments from subjects using stimuli from the correspondence detection task in order to more directly compare these paradigms for investigating multisensory processing.

Experiments 2 and 3 went further in showing that increased temporal pattern *complexity* (higher entropy or unpredictability) improved detection of audio-visual correspondence. Participants were more sensitive to audio-visual correspondences at all lags when the stimulus streams were random (with higher pattern complexity, as quantified by ISI entropy) than when the streams had rhythmic periodicities (resulting in lower pattern complexity). Experiment 3 showed that the second-order entropy of a stream’s ISIs (perhaps along with higher-order entropies) was sufficient to drive enhanced correspondence detection when lower-order entropy was controlled. That is, observers benefited from increases in the information contained in the temporal *sequence* of ISIs, when the variability of ISIs was identical. Two temporally complex, unpredictable stimulus streams are less likely to have the same temporal pattern by chance than two predictable streams, thereby providing stronger cues that, when matching, they are likely to have a common external cause. The importance of common temporal structure in multisensory integration has recently been demonstrated by Parise et al. (2012) using a spatial localization task. Their study showed that location information from auditory and visual stimuli was combined optimally only when the stimuli had identical temporal patterns. This suggests that temporal correlation cues the brain to attribute a common cause to input from different modalities. Our results demonstrate that people are sensitive to specific aspects of temporal information in multisensory stimulus streams and can use it to detect corresponding temporal patterns across modalities. These results are in line with proposals that generative models may guide inferences about likely external causes for sensory impressions (Rao and Ballard, 1999; Friston, 2005).

On the other hand, the observed enhancement in correspondence sensitivity with higher stream complexity goes against an intuitive possibility for how people might match auditory and visual streams, namely by comparing sequences from each modality stored in short-term memory. Such an explicitly mnemonic process should be facilitated with predictable/rhythmic streams, rather than more complex patterns as we found, since the latter are less redundant and thus harder to remember or reproduce. Our results support instead a more statistical perspective on pattern-matching of complex multisensory streams, whereby patterns of higher irregularity lead to better performance because they contain more temporal information. A similar framework has been proposed for grouping in the visual domain, with Lee and Blake (1999) showing enhanced spatial grouping for elements changing according to a matching, high-entropy temporal pattern. The entropy measure used by Lee and Blake corresponds to what we have called event entropy, which was controlled in our experiments. We instead tested observers’ sensitivity to the entropy of the distribution (Experiment 2) and sequence (Experiment 3) of inter-event intervals, which captures higher-order properties of temporal structure. This information theory-based approach may offer a formal handle for studying the role of distinctive audio-visual patterns in multisensory perception (Fujisaki and Nishida, 2005, 2007, 2008), which may be especially relevant for understanding multisensory perception under natural conditions.

We used information-theoretic measures of entropy to characterize different aspects of the temporal complexity of our stimulus streams. This approach had the advantages of providing precise, formal descriptions of complexity and of connecting to other formal work on information processing in sensory systems (Rieke et al., 1997). However, although we have characterized the complexity of our stimuli when considered as binary event streams, inter-event intervals, or ordered sequences of intervals, there may be other ways of describing our stimuli that we have not explored here. Many naturally occurring auditory and visual stimuli have a temporal structure that can be described in terms of the timing of salient events or in terms of successive temporal intervals – speech, with its syllabic temporal structure, is one example. Our formal methods are well suited to characterizing the temporal complexity of stimuli with these properties, but they are less appropriate for characterizing more continuously varying stimuli that lack event or interval structure. In addition, the use of information theory to measure temporal information in simple sensory stimuli allowed us to uncover different aspects of complexity that influenced human perception; however, it does not imply that the human brain computes these specific information-theoretic quantities when performing the task. Future work will be required to model and test for the neural mechanisms underlying the sensitivity to temporal complexity described here.

Cook et al. (2011) recently compared synchrony perception for tones and flashes that were equally spaced in time but appeared in either a predictable order (alternating high and low tones in two auditory streams which were simultaneously ascending or descending in pitch) or a less predictable order (a scrambled version of this sequence). They found that observers perceived audio-visual synchrony over wider time windows for the more unpredictable streams. Wider windows for audio-visual synchrony perception have also been found for certain complex stimuli, specifically music and action videos, when played in the reverse as compared to the forward direction (Vatakis and Spence, 2006). Each of these findings can be interpreted in the context of predictability: audio-visual stimuli with less predictable event ordering are associated with wider time windows for audio-visual synchrony perception. However, it is sometimes unclear how to interpret a widening in synchrony perception time windows. On the one hand, wider time windows may suggest more permissive audio-visual binding, and in this sense more effective multisensory integration. On the other hand, wider time windows may entail reduced accuracy on synchrony detection tasks (poorer synchrony/asynchrony discrimination), and so may indicate less effective multisensory integration. Our matching task results help to resolve such difficulties in interpreting the effects of predictability on synchrony perception by showing an *improvement* in behavioral performance for more complex and thus less predictable stimuli. Further, a simple widening of synchrony perception windows would be unlikely to result in performance enhancements in the matching task, since wide temporal windows would encompass both matching and non-matching stimulus events. Instead, our results reflect the observers’ sensitivity to temporal *pattern* in the perception of multisensory relationships and show that changes in the predictability of temporal intervals, in the absence of other forms of stimulus complexity or variability, is sufficient to drive changes in the perception of audio-visual correspondence.

Recent neuroimaging studies have already shown that auditory cortex is sensitive to the temporal complexity of pitch sequences (Overath et al., 2007) and that audio-visual multisensory interactions can affect even primary auditory and visual cortices when using complex, related audio-visual streams, similar to those used in the present study (Noesselt et al., 2007). Future work with neural measures could test the dependence of such activations on audio-visual lag and stream complexity, analogously to the psychophysical manipulations introduced here. Finally, in providing simple objective tests for sensitivity to audio-visual correspondence in the temporal patterning of stimuli, the new paradigms developed here could be particularly useful for future application to the study of disorders hypothesized to involve abnormalities in multisensory aspects of temporal pattern processing [e.g., developmental conditions such as dyslexia (Goswami, 2011)].

## Conflict of Interest Statement

The authors declare that the research was conducted in the absence of any commercial or financial relationships that could be construed as a potential conflict of interest.
